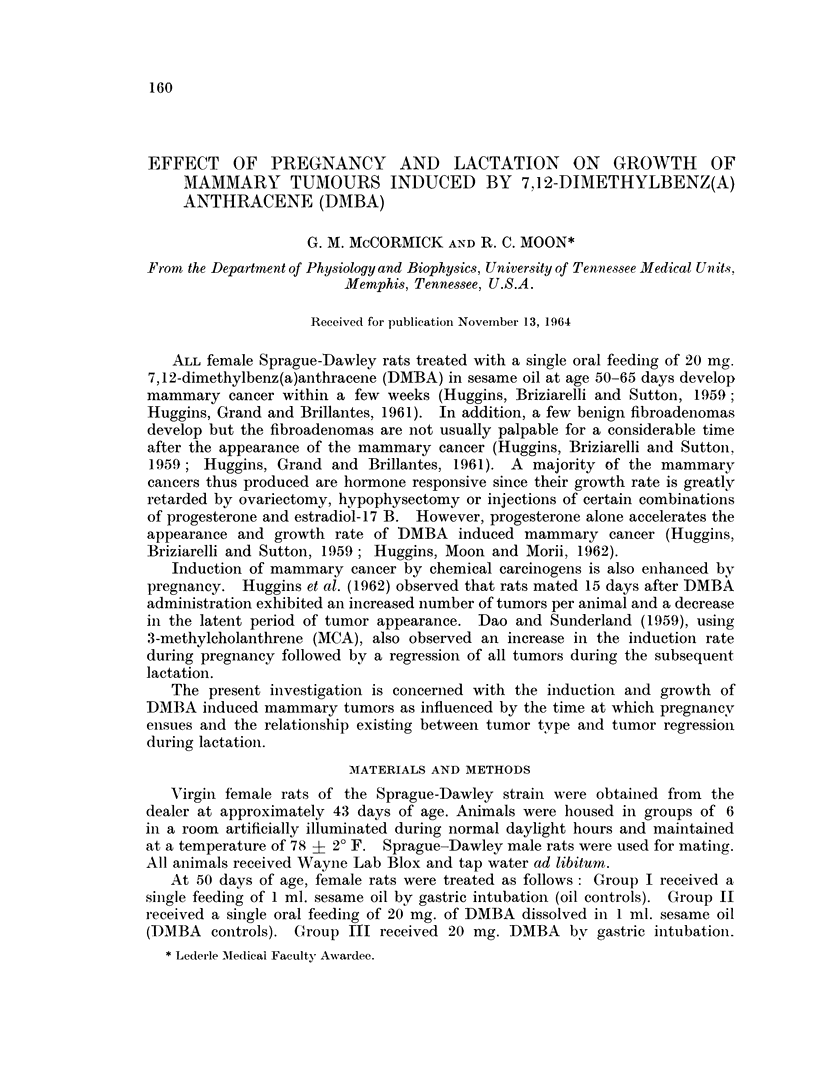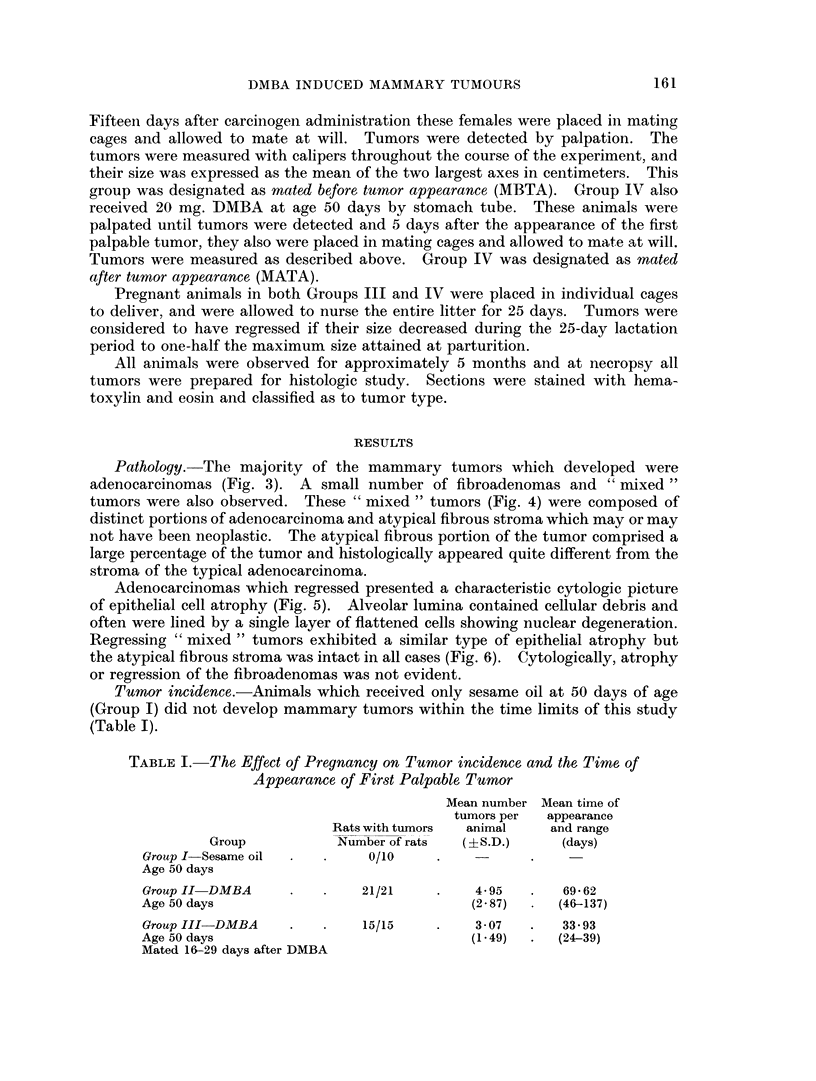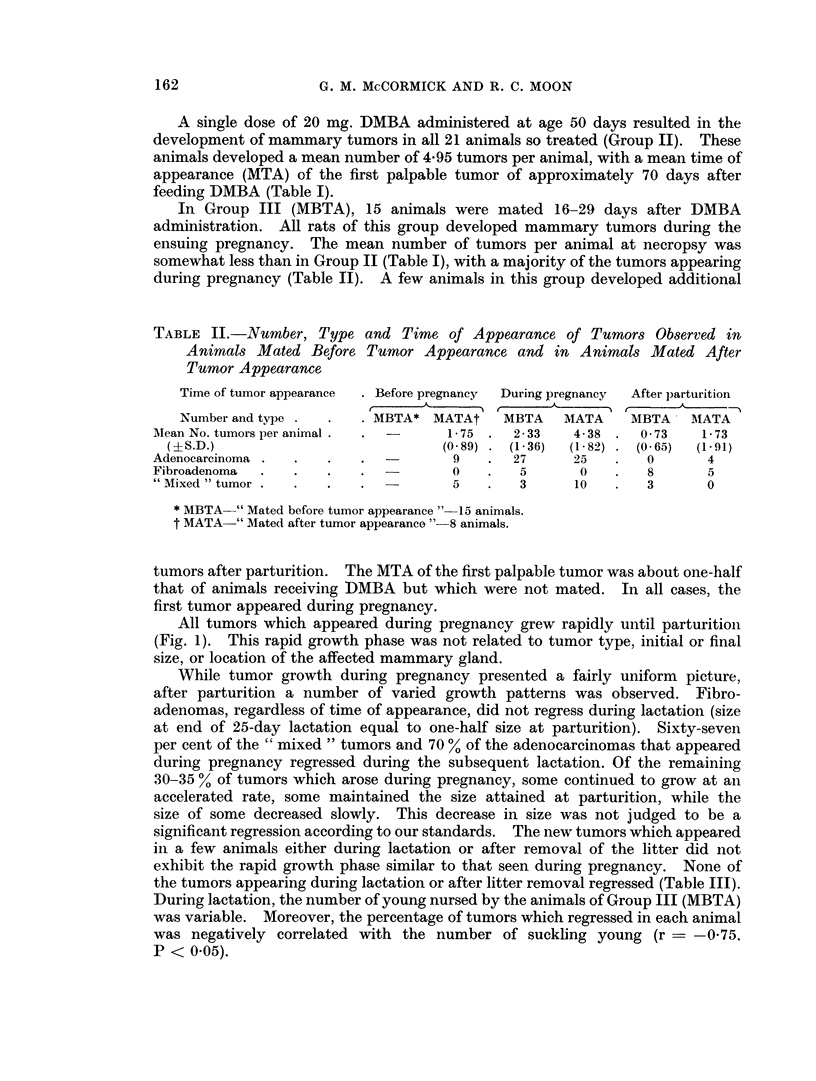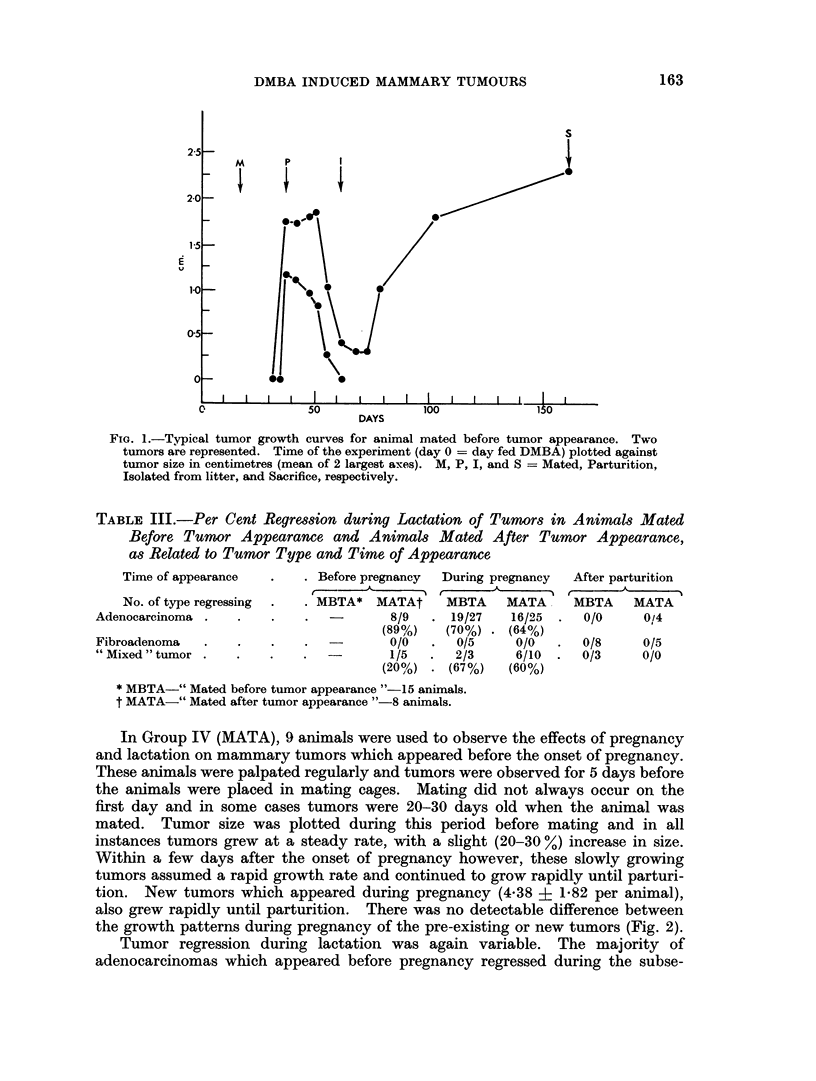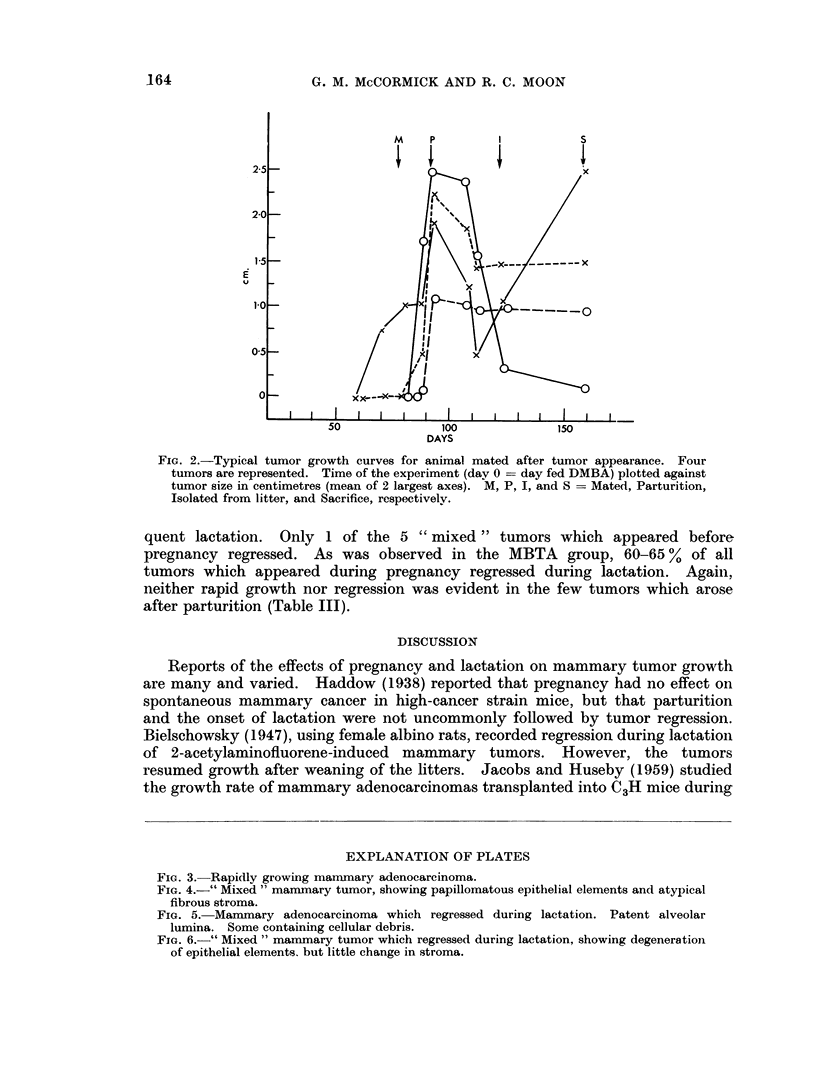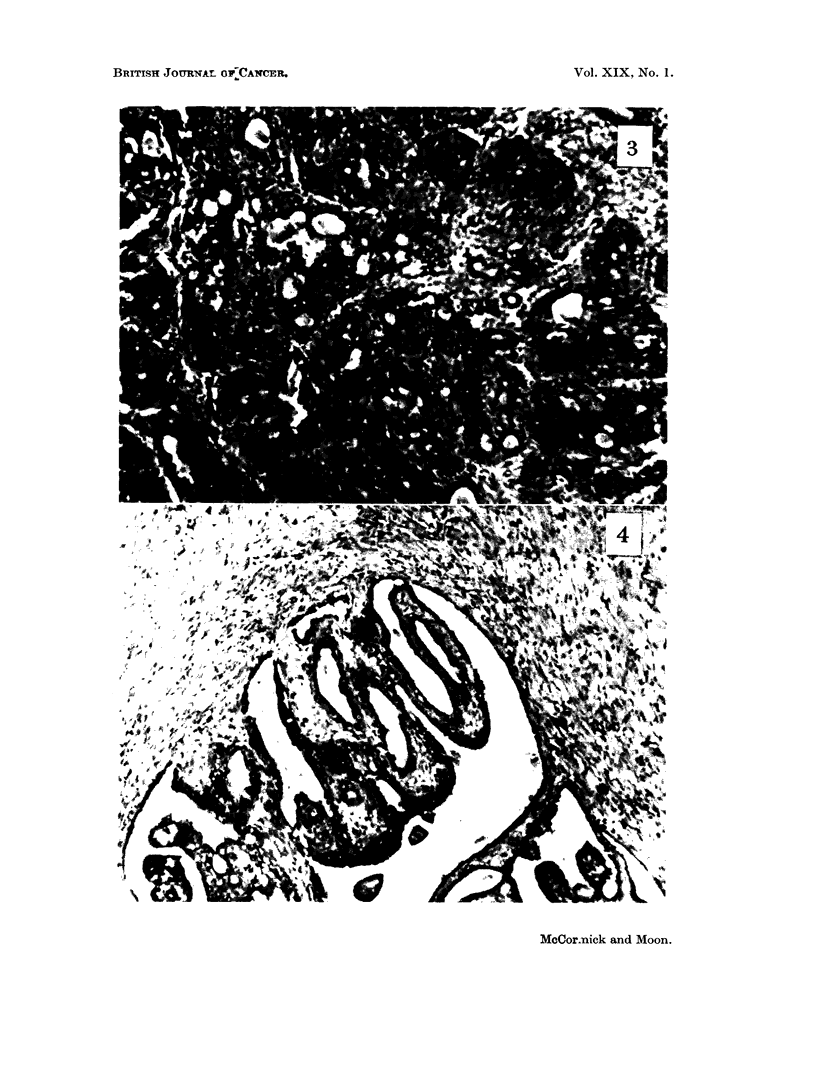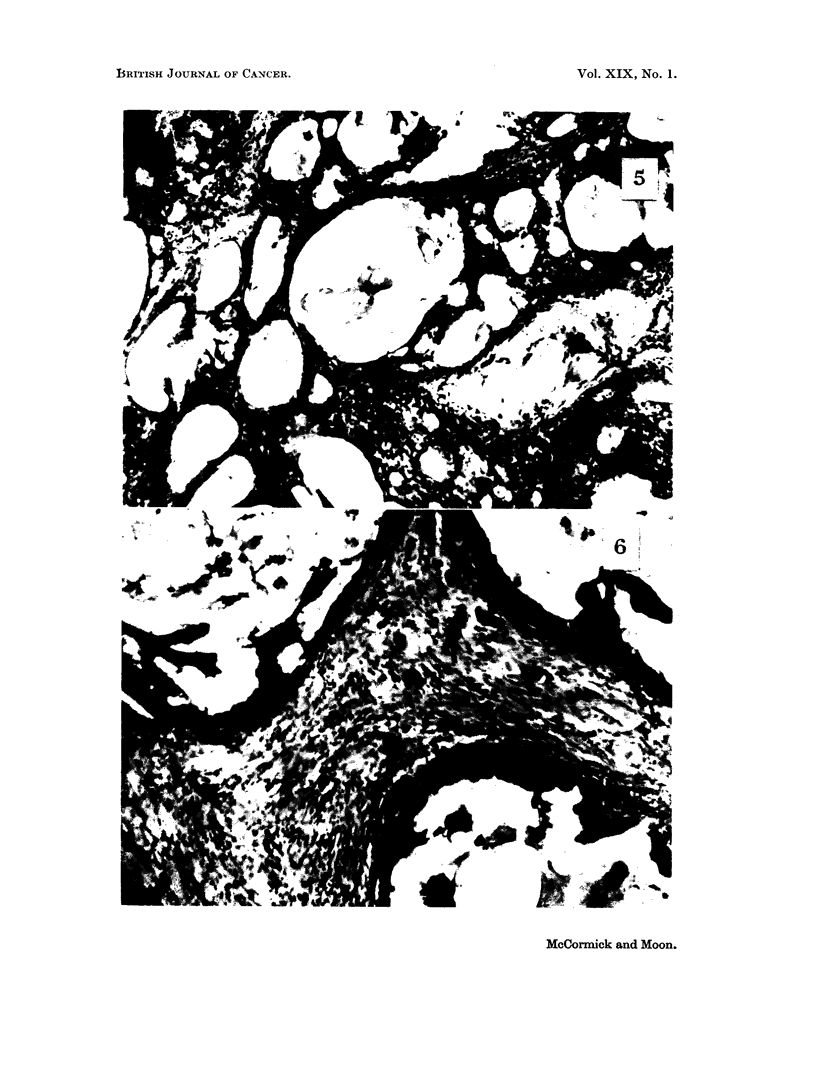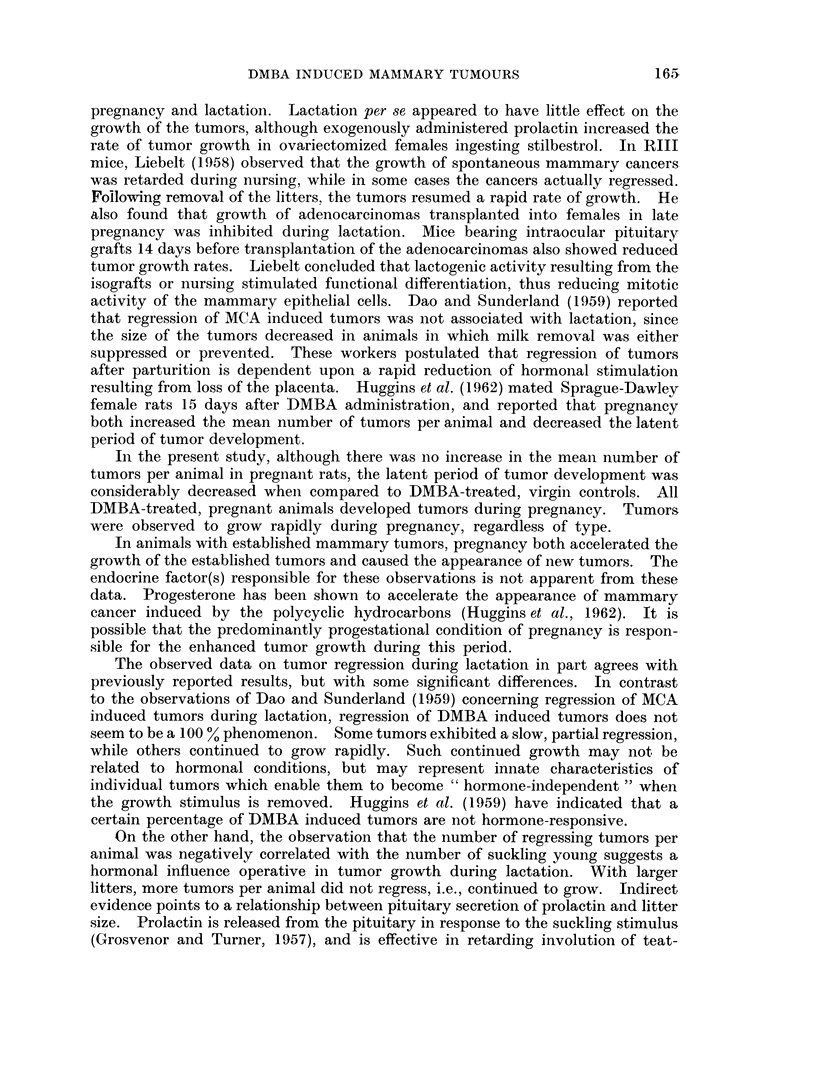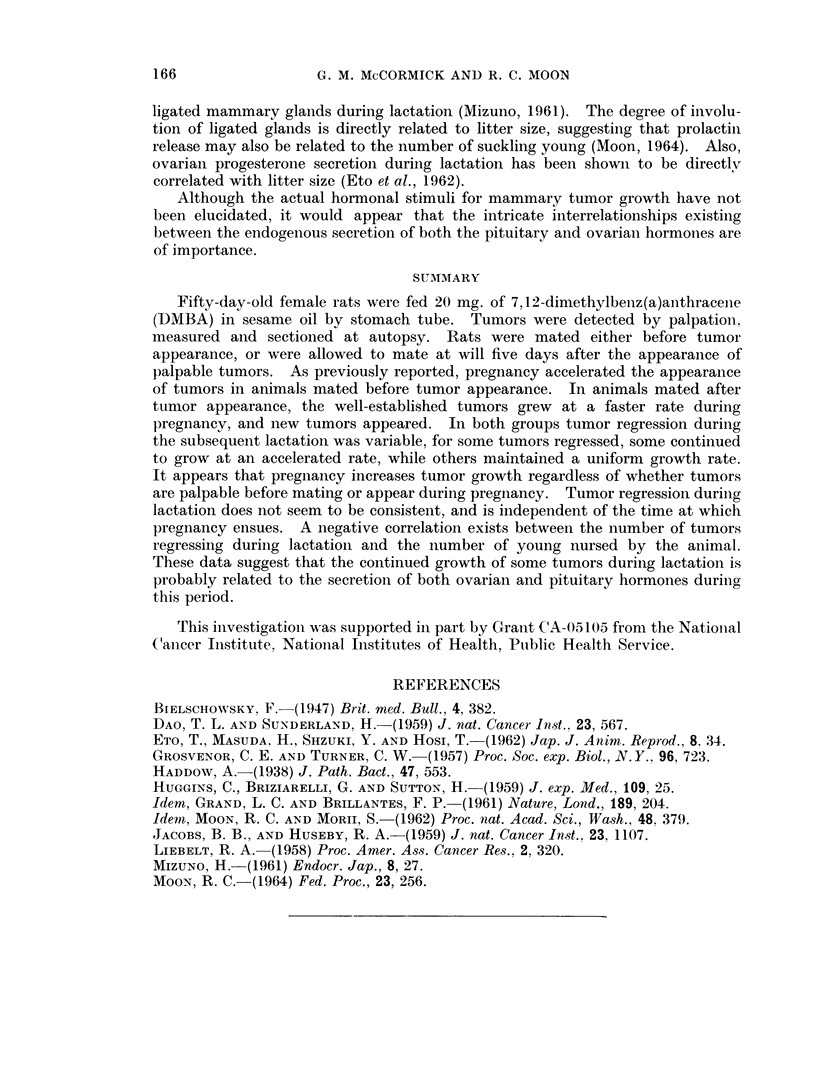# Effect of Pregnancy and Lactation on Growth of Mammary Tumours Induced by 7,12-Dimethylbenz(a)anthracene (DMBA)

**DOI:** 10.1038/bjc.1965.19

**Published:** 1965-03

**Authors:** G. M. McCormick, R. C. Moon

## Abstract

**Images:**


### EFFECT OF PREGNANCY AND LACTATION ON GROWTH OF MAMMARY TUMOURS INDUCED BY 7,12-DIMETHYLBENZ(A) ANTHRACENE (DMBA)

G. M. McCORMICK AND R. C. MOON*

*From the Department of Physiology and Biophysics, University of Tennessee Medical Units, Memphis, Tennessee, U.S.A.*

Received for publication November 13, 1964

ALL female Sprague-Dawley rats treated with a single oral feeding of 20 mg. 7,12-dimethylbenz(a)anthracene (DMBA) in sesame oil at age 50–65 days develop mammary cancer within a few weeks (Huggins, Briziarelli and Sutton, 1959; Huggins, Grand and Brillantes, 1961). In addition, a few benign fibroadenomas develop but the fibroadenomas are not usually palpable for a considerable time after the appearance of the mammary cancer (Huggins, Briziarelli and Sutton, 1959; Huggins, Grand and Brillantes, 1961). A majority of the mammary cancers thus produced are hormone responsive since their growth rate is greatly retarded by ovariectomy, hypophysectomy or injections of certain combinations of progesterone and estradiol-17 B. However, progesterone alone accelerates the appearance and growth rate of DMBA induced mammary cancer (Huggins, Briziarelli and Sutton, 1959; Huggins, Moon and Morii, 1962).

Induction of mammary cancer by chemical carcinogens is also enhanced by pregnancy. Huggins *et al.* (1962) observed that rats mated 15 days after DMBA administration exhibited an increased number of tumors per animal and a decrease in the latent period of tumor appearance. Dao and Sunderland (1959), using 3-methylcholanthrene (MCA), also observed an increase in the induction rate during pregnancy followed by a regression of all tumors during the subsequent lactation.

The present investigation is concerned with the induction and growth of DMBA induced mammary tumors as influenced by the time at which pregnancy ensues and the relationship existing between tumor type and tumor regression during lactation.

### MATERIALS AND METHODS

Virgin female rats of the Sprague-Dawley strain were obtained from the dealer at approximately 43 days of age. Animals were housed in groups of 6 in a room artificially illuminated during normal daylight hours and maintained at a temperature of 78 $\pm$ 2° F. Sprague–Dawley male rats were used for mating. All animals received Wayne Lab Blox and tap water *ad libitum.*

At 50 days of age, female rats were treated as follows: Group I received a single feeding of 1 ml. sesame oil by gastric intubation (oil controls). Group II received a single oral feeding of 20 mg. of DMBA dissolved in 1 ml. sesame oil (DMBA controls). Group III received 20 mg. DMBA by gastric intubation.

* Lederle Medical Faculty Awardee.

Fifteen days after carcinogen administration these females were placed in mating cages and allowed to mate at will. Tumors were detected by palpation. The tumors were measured with calipers throughout the course of the experiment, and their size was expressed as the mean of the two largest axes in centimeters. This group was designated as *mated before tumor appearance* (MBTA). Group IV also received 20 mg. DMBA at age 50 days by stomach tube. These animals were palpated until tumors were detected and 5 days after the appearance of the first palpable tumor, they also were placed in mating cages and allowed to mate at will. Tumors were measured as described above. Group IV was designated as *mated after tumor appearance* (MATA).

Pregnant animals in both Groups III and IV were placed in individual cages to deliver, and were allowed to nurse the entire litter for 25 days. Tumors were considered to have regressed if their size decreased during the 25-day lactation period to one-half the maximum size attained at parturition.

All animals were observed for approximately 5 months and at necropsy all tumors were prepared for histologic study. Sections were stained with hematoxylin and eosin and classified as to tumor type.

### RESULTS

*Pathology.*—The majority of the mammary tumors which developed were adenocarcinomas (Fig. 3). A small number of fibroadenomas and " mixed " tumors were also observed. These " mixed " tumors (Fig. 4) were composed of distinct portions of adenocarcinoma and atypical fibrous stroma which may or may not have been neoplastic. The atypical fibrous portion of the tumor comprised a large percentage of the tumor and histologically appeared quite different from the stroma of the typical adenocarcinoma.

Adenocarcinomas which regressed presented a characteristic cytologic picture of epithelial cell atrophy (Fig. 5). Alveolar lumina contained cellular debris and often were lined by a single layer of flattened cells showing nuclear degeneration. Regressing " mixed " tumors exhibited a similar type of epithelial atrophy but the atypical fibrous stroma was intact in all cases (Fig. 6). Cytologically, atrophy or regression of the fibroadenomas was not evident.

*Tumor incidence.*—Animals which received only sesame oil at 50 days of age (Group I) did not develop mammary tumors within the time limits of this study (Table I).

TABLE I.—*The Effect of Pregnancy on Tumor incidence and the Time of Appearance of First Palpable Tumor*

| Group | Rats with tumors / Number of rats | Mean number tumors per animal (±S.D.) | Mean time of appearance and range (days) |
|---|---|---|---|
| *Group I*—Sesame oil<br>Age 50 days | 0/10 | — | — |
| *Group II—DMBA*<br>Age 50 days | 21/21 | 4·95<br>(2·87) | 69·62<br>(46–137) |
| *Group III—DMBA*<br>Age 50 days<br>Mated 16–29 days after DMBA | 15/15 | 3·07<br>(1·49) | 33·93<br>(24–39) |

A single dose of 20 mg. DMBA administered at age 50 days resulted in the development of mammary tumors in all 21 animals so treated (Group II). These animals developed a mean number of 4·95 tumors per animal, with a mean time of appearance (MTA) of the first palpable tumor of approximately 70 days after feeding DMBA (Table I).

In Group III (MBTA), 15 animals were mated 16–29 days after DMBA administration. All rats of this group developed mammary tumors during the ensuing pregnancy. The mean number of tumors per animal at necropsy was somewhat less than in Group II (Table I), with a majority of the tumors appearing during pregnancy (Table II). A few animals in this group developed additional

TABLE II.—*Number, Type and Time of Appearance of Tumors Observed in Animals Mated Before Tumor Appearance and in Animals Mated After Tumor Appearance*

| Time of tumor appearance | Before pregnancy | | During pregnancy | | After parturition | |
|---|---|---|---|---|---|---|
| Number and type | MBTA* | MATA† | MBTA | MATA | MBTA | MATA |
| Mean No. tumors per animal | — | 1·75 | 2·33 | 4·38 | 0·73 | 1·73 |
| (±S.D.) | | (0·89) | (1·36) | (1·82) | (0·65) | (1·91) |
| Adenocarcinoma | — | 9 | 27 | 25 | 0 | 4 |
| Fibroadenoma | — | 0 | 5 | 0 | 8 | 5 |
| " Mixed " tumor | — | 5 | 3 | 10 | 3 | 0 |

* MBTA—" Mated before tumor appearance "—15 animals.
† MATA—" Mated after tumor appearance "—8 animals.

tumors after parturition. The MTA of the first palpable tumor was about one-half that of animals receiving DMBA but which were not mated. In all cases, the first tumor appeared during pregnancy.

All tumors which appeared during pregnancy grew rapidly until parturition (Fig. 1). This rapid growth phase was not related to tumor type, initial or final size, or location of the affected mammary gland.

While tumor growth during pregnancy presented a fairly uniform picture, after parturition a number of varied growth patterns was observed. Fibroadenomas, regardless of time of appearance, did not regress during lactation (size at end of 25-day lactation equal to one-half size at parturition). Sixty-seven per cent of the " mixed " tumors and 70 % of the adenocarcinomas that appeared during pregnancy regressed during the subsequent lactation. Of the remaining 30–35 % of tumors which arose during pregnancy, some continued to grow at an accelerated rate, some maintained the size attained at parturition, while the size of some decreased slowly. This decrease in size was not judged to be a significant regression according to our standards. The new tumors which appeared in a few animals either during lactation or after removal of the litter did not exhibit the rapid growth phase similar to that seen during pregnancy. None of the tumors appearing during lactation or after litter removal regressed (Table III). During lactation, the number of young nursed by the animals of Group III (MBTA) was variable. Moreover, the percentage of tumors which regressed in each animal was negatively correlated with the number of suckling young ($r = -0{\cdot}75$, $P < 0{\cdot}05$).

FIG. 1.—Typical tumor growth curves for animal mated before tumor appearance. Two tumors are represented. Time of the experiment (day 0 = day fed DMBA) plotted against tumor size in centimetres (mean of 2 largest axes). M, P, I, and S = Mated, Parturition, Isolated from litter, and Sacrifice, respectively.

TABLE III.—*Per Cent Regression during Lactation of Tumors in Animals Mated Before Tumor Appearance and Animals Mated After Tumor Appearance, as Related to Tumor Type and Time of Appearance*

| Time of appearance | Before pregnancy | | During pregnancy | | After parturition | |
|---|---|---|---|---|---|---|
| No. of type regressing | MBTA* | MATA† | MBTA | MATA | MBTA | MATA |
| Adenocarcinoma | — | 8/9 (89%) | 19/27 (70%) | 16/25 (64%) | 0/0 | 0/4 |
| Fibroadenoma | — | 0/0 | 0/5 | 0/0 | 0/8 | 0/5 |
| " Mixed " tumor | — | 1/5 (20%) | 2/3 (67%) | 6/10 (60%) | 0/3 | 0/0 |

* MBTA—" Mated before tumor appearance "—15 animals.
† MATA—" Mated after tumor appearance "—8 animals.

In Group IV (MATA), 9 animals were used to observe the effects of pregnancy and lactation on mammary tumors which appeared before the onset of pregnancy. These animals were palpated regularly and tumors were observed for 5 days before the animals were placed in mating cages. Mating did not always occur on the first day and in some cases tumors were 20–30 days old when the animal was mated. Tumor size was plotted during this period before mating and in all instances tumors grew at a steady rate, with a slight (20–30%) increase in size. Within a few days after the onset of pregnancy however, these slowly growing tumors assumed a rapid growth rate and continued to grow rapidly until parturition. New tumors which appeared during pregnancy (4·38 ± 1·82 per animal), also grew rapidly until parturition. There was no detectable difference between the growth patterns during pregnancy of the pre-existing or new tumors (Fig. 2).

Tumor regression during lactation was again variable. The majority of adenocarcinomas which appeared before pregnancy regressed during the subse-

FIG. 2.—Typical tumor growth curves for animal mated after tumor appearance. Four tumors are represented. Time of the experiment (day 0 = day fed DMBA) plotted against tumor size in centimetres (mean of 2 largest axes). M, P, I, and S = Mated, Parturition, Isolated from litter, and Sacrifice, respectively.

quent lactation. Only 1 of the 5 "mixed" tumors which appeared before pregnancy regressed. As was observed in the MBTA group, 60–65% of all tumors which appeared during pregnancy regressed during lactation. Again, neither rapid growth nor regression was evident in the few tumors which arose after parturition (Table III).

### DISCUSSION

Reports of the effects of pregnancy and lactation on mammary tumor growth are many and varied. Haddow (1938) reported that pregnancy had no effect on spontaneous mammary cancer in high-cancer strain mice, but that parturition and the onset of lactation were not uncommonly followed by tumor regression. Bielschowsky (1947), using female albino rats, recorded regression during lactation of 2-acetylaminofluorene-induced mammary tumors. However, the tumors resumed growth after weaning of the litters. Jacobs and Huseby (1959) studied the growth rate of mammary adenocarcinomas transplanted into $C_3H$ mice during

---

### EXPLANATION OF PLATES

FIG. 3.—Rapidly growing mammary adenocarcinoma.

FIG. 4.—" Mixed " mammary tumor, showing papillomatous epithelial elements and atypical fibrous stroma.

FIG. 5.—Mammary adenocarcinoma which regressed during lactation. Patent alveolar lumina. Some containing cellular debris.

FIG. 6.—" Mixed " mammary tumor which regressed during lactation, showing degeneration of epithelial elements, but little change in stroma.

McCormick and Moon.

5

6

McCormick and Moon.

pregnancy and lactation. Lactation *per se* appeared to have little effect on the growth of the tumors, although exogenously administered prolactin increased the rate of tumor growth in ovariectomized females ingesting stilbestrol. In RIII mice, Liebelt (1958) observed that the growth of spontaneous mammary cancers was retarded during nursing, while in some cases the cancers actually regressed. Following removal of the litters, the tumors resumed a rapid rate of growth. He also found that growth of adenocarcinomas transplanted into females in late pregnancy was inhibited during lactation. Mice bearing intraocular pituitary grafts 14 days before transplantation of the adenocarcinomas also showed reduced tumor growth rates. Liebelt concluded that lactogenic activity resulting from the isografts or nursing stimulated functional differentiation, thus reducing mitotic activity of the mammary epithelial cells. Dao and Sunderland (1959) reported that regression of MCA induced tumors was not associated with lactation, since the size of the tumors decreased in animals in which milk removal was either suppressed or prevented. These workers postulated that regression of tumors after parturition is dependent upon a rapid reduction of hormonal stimulation resulting from loss of the placenta. Huggins *et al.* (1962) mated Sprague-Dawley female rats 15 days after DMBA administration, and reported that pregnancy both increased the mean number of tumors per animal and decreased the latent period of tumor development.

In the present study, although there was no increase in the mean number of tumors per animal in pregnant rats, the latent period of tumor development was considerably decreased when compared to DMBA-treated, virgin controls. All DMBA-treated, pregnant animals developed tumors during pregnancy. Tumors were observed to grow rapidly during pregnancy, regardless of type.

In animals with established mammary tumors, pregnancy both accelerated the growth of the established tumors and caused the appearance of new tumors. The endocrine factor(s) responsible for these observations is not apparent from these data. Progesterone has been shown to accelerate the appearance of mammary cancer induced by the polycyclic hydrocarbons (Huggins *et al.*, 1962). It is possible that the predominantly progestational condition of pregnancy is responsible for the enhanced tumor growth during this period.

The observed data on tumor regression during lactation in part agrees with previously reported results, but with some significant differences. In contrast to the observations of Dao and Sunderland (1959) concerning regression of MCA induced tumors during lactation, regression of DMBA induced tumors does not seem to be a 100 % phenomenon. Some tumors exhibited a slow, partial regression, while others continued to grow rapidly. Such continued growth may not be related to hormonal conditions, but may represent innate characteristics of individual tumors which enable them to become " hormone-independent " when the growth stimulus is removed. Huggins *et al.* (1959) have indicated that a certain percentage of DMBA induced tumors are not hormone-responsive.

On the other hand, the observation that the number of regressing tumors per animal was negatively correlated with the number of suckling young suggests a hormonal influence operative in tumor growth during lactation. With larger litters, more tumors per animal did not regress, i.e., continued to grow. Indirect evidence points to a relationship between pituitary secretion of prolactin and litter size. Prolactin is released from the pituitary in response to the suckling stimulus (Grosvenor and Turner, 1957), and is effective in retarding involution of teatligated mammary glands during lactation (Mizuno, 1961). The degree of involution of ligated glands is directly related to litter size, suggesting that prolactin release may also be related to the number of suckling young (Moon, 1964). Also, ovarian progesterone secretion during lactation has been shown to be directly correlated with litter size (Eto *et al.*, 1962).

Although the actual hormonal stimuli for mammary tumor growth have not been elucidated, it would appear that the intricate interrelationships existing between the endogenous secretion of both the pituitary and ovarian hormones are of importance.

SUMMARY

Fifty-day-old female rats were fed 20 mg. of 7,12-dimethylbenz(a)anthracene (DMBA) in sesame oil by stomach tube. Tumors were detected by palpation, measured and sectioned at autopsy. Rats were mated either before tumor appearance, or were allowed to mate at will five days after the appearance of palpable tumors. As previously reported, pregnancy accelerated the appearance of tumors in animals mated before tumor appearance. In animals mated after tumor appearance, the well-established tumors grew at a faster rate during pregnancy, and new tumors appeared. In both groups tumor regression during the subsequent lactation was variable, for some tumors regressed, some continued to grow at an accelerated rate, while others maintained a uniform growth rate. It appears that pregnancy increases tumor growth regardless of whether tumors are palpable before mating or appear during pregnancy. Tumor regression during lactation does not seem to be consistent, and is independent of the time at which pregnancy ensues. A negative correlation exists between the number of tumors regressing during lactation and the number of young nursed by the animal. These data suggest that the continued growth of some tumors during lactation is probably related to the secretion of both ovarian and pituitary hormones during this period.

This investigation was supported in part by Grant CA-05105 from the National Cancer Institute, National Institutes of Health, Public Health Service.

REFERENCES

Bielschowsky, F.—(1947) *Brit. med. Bull.*, **4**, 382.
Dao, T. L. and Sunderland, H.—(1959) *J. nat. Cancer Inst.*, **23**, 567.
Eto, T., Masuda, H., Shzuki, Y. and Hosi, T.—(1962) *Jap. J. Anim. Reprod.*, **8**, 34.
Grosvenor, C. E. and Turner, C. W.—(1957) *Proc. Soc. exp. Biol., N.Y.*, **96**, 723.
Haddow, A.—(1938) *J. Path. Bact.*, **47**, 553.
Huggins, C., Briziarelli, G. and Sutton, H.—(1959) *J. exp. Med.*, **109**, 25.
*Idem*, Grand, L. C. and Brillantes, F. P.—(1961) *Nature, Lond.*, **189**, 204.
*Idem*, Moon, R. C. and Morii, S.—(1962) *Proc. nat. Acad. Sci., Wash.*, **48**, 379.
Jacobs, B. B., and Huseby, R. A.—(1959) *J. nat. Cancer Inst.*, **23**, 1107.
Liebelt, R. A.—(1958) *Proc. Amer. Ass. Cancer Res.*, **2**, 320.
Mizuno, H.—(1961) *Endocr. Jap.*, **8**, 27.
Moon, R. C.—(1964) *Fed. Proc.*, **23**, 256.